# The Impostor Phenomenon: Toward a Better Understanding of the Nomological Network and Gender Differences

**DOI:** 10.3389/fpsyg.2021.764030

**Published:** 2021-11-17

**Authors:** Monika Fleischhauer, Josephine Wossidlo, Lars Michael, Sören Enge

**Affiliations:** Department of Psychology, Faculty of Natural Sciences, MSB Medical School Berlin, Berlin, Germany

**Keywords:** impostor phenomenon, sandbagging, perfectionism, gender effects, strategic behavior, personality correlates, discriminant validity

## Abstract

The impostor phenomenon (IP) refers to the tendency to perceive oneself as intellectually incompetent and to attribute one’s own success to effort-related or external factors, such as fortunate circumstances. The present study (*N*=209) aimed to contribute to open questions regarding gender differences in the IP and the nomological network of the IP. The results show that the consistently found key correlates of the IP, that is, lower self-esteem and higher neuroticism, could also play a role in explaining why women report higher impostor feelings than men in many studies. Moreover, the results suggest that IP is characterized by the more maladaptive, socially prescribed perfectionism, which is related to the belief that others expect perfection from oneself, whereas self-oriented perfectionism, which is characterized by a critical view on oneself, plays a smaller role in differences in the IP. Finally, a strong association with the sandbagging construct challenges the conceptualization of the IP as a genuine doubt about one’s own competence, because similarly to IP, sandbaggers present themselves negatively to others, but do so for very strategic reasons in order to create a low expectation base in other individuals. Regression analysis was used to assess the incremental value of the personality factors in explaining variance in the IP. It was found that sandbagging and IP are highly related but not interchangeable.

## The Impostor Phenomenon

The term impostor phenomenon (IP) was first introduced in 1978 by Clance and Imes and denotes an inner experience of intellectual inadequacy that is accompanied by a strongly limited confidence in own abilities, despite that intellectual achievements (e.g., at the workplace or in the academic field) objectively mirror the opposite. Instead of attributing academic or work-related success to own abilities, people with impostor feelings tend to see external or effort-related factors such as fortunate circumstances, knowing the right people or hard work as the original cause of their success (e.g., Clance and OToole, 1987). Therefore, they are convinced not to deserve their achievements and thus cannot really accept the appreciation and respect from others. In fact, they believe that they have mislead other people regarding their abilities and capabilities, and they are afraid that their supposed incompetence will eventually be revealed (e.g., Leary et al., 2000).

Clance and Imes (1978) observed that a selected cohort of professionally and academically high achieving women suffered from extreme fears of failure and therefore expected that the IP would especially be experienced by women. As indicated by a systematic review of Bravata et al. (2019) summarizing the results of thirty-three studies on this issue, a mixed result pattern becomes apparent. While half of the studies support gender-specific differences in the IP with small (e.g., Jöstl et al., 2015; Patzak et al., 2017) to moderate effect sizes (e.g., Hayes and Davis, 1993; McGregor et al., 2008), the other half found no differences between woman and men (e.g., Leonhardt et al., 2017). Given these mixed results, it seems important and was the aim of the present study to better understand the processes behind gender differences in the IP. This could also help to identify contextual factors that may lead to gender differences in the IP. For example, gender-specific differences in the IP might particularly occur in the late adolescence, where females tend to have a lower self-esteem than men (see Zuckerman et al., 2016), and thus might experience more impostor feelings. As lower self-esteem is deemed to be a core correlate of the IP (e.g., Chrisman et al., 1995; Schubert and Bowker, 2019), it could modulate gender-specific differences in the IP.

Several studies dealing with construct validation issues suggest that high neuroticism or anxiety and low self-esteem are personality-related key correlates of the IP (e.g., Thompson et al., 2000; Vergauwe et al., 2015; Schubert and Bowker, 2019). In a similar vein, Ross and Krukowski (2003), who examined IP in the context of personality pathology, defined IP as a maladaptive personality style that is related to fear, inferiority, detachment, and self-deprecation.

Furthermore, several studies emphasize that perfectionism is a key correlate of impostor feelings (Thompson et al., 2000; Ferrari and Thompson, 2006; Dudău, 2014; Vergauwe et al., 2015; Rohrmann et al., 2016). In a sample of employees, Vergauwe et al. (2015) showed that the IP is closely related to maladaptive perfectionism that is characterized by the setting of unattainably high goals and the inability to rejoice in one’s own performance. These results have been largely replicated by Rohrmann et al. (2016) in a cohort of managers. In another prominent concept of Hewitt and Flett (1991), three forms of perfectionism are distinguished: self-oriented perfectionism, socially prescribed perfectionism, and others-oriented perfectionism, differing with regard to the convictions of the perfectionists. Self-oriented perfectionists have a very high personal standard and are extremely self-critical if they do not fulfil their own expectations. In comparison, socially prescribed perfectionism includes the belief that others expect perfection from oneself. While other-oriented perfectionism is about high expectations of others, self-oriented and socially prescribed perfectionism relate to the self and might be of particular interest with regard to IP. More specifically, the question of whether the inner experience of intellectual inadequacy is related to an excessively high personal aspiration or rather to presumed expectations of others could help to learn more about the underlying motives of the negative self-representations of individuals with impostor feelings.

Originally, it was assumed that the supposed fear of failure of an impostor resulted from a negative self-assessment (Clance and Imes, 1978), as outlined above. However, another line of research has shown that people tend to present themselves in an unfavorable light if they believe that negative self-presentation has a social value for them (e.g., Baumeister et al., 1979; Leary et al., 2000). Leary et al. (2000) assumed that public self-accusation cannot be both a strategy and an essential facet of the impostor. They hypothesized that different types could be distinguished within the IP: Individuals who really see themselves as incompetent and others whose negative self-presentation is primarily strategically motivated. Support for this assumption was found by Leonhardt et al. (2017) by means of a cluster analysis in a sample of managers. The so-called “true impostors” were characterized by high levels of anxiety, negative self-evaluations, perfectionism, and the experience of work-related stress. For the other cluster, the so-called “strategic impostors,” these impostor-related personality correlates were significantly less pronounced and indicators of positive self-evaluation were more pronounced. However, Leary et al. (2000) did not find empirical support for different types of impostors. According to their result that individuals with high impostor feelings only tended to a negative self-presentation when their performance was public, the authors concluded that negative self-evaluation was more likely to follow the goal of gaining interpersonal advantages. To further follow the assumption of a rather strategic self-presentation, the relationship of IP and *sandbagging* could be investigated. Sandbagging refers to a self-presentational strategy and describes a behavior of negating one’s own effort and making false claims of supposed incompetence (Gibson and Sachau, 2000; Gibson et al., 2002; Petersen, 2014). The term sandbagging is used in competitive settings, for example, when card players have a good hand but pretend to have a bad one. Sandbagging behavior was, however, also shown in non-competing but evaluative contexts, such as academic ones, for example, when well-prepared students claim not to expect good results in an exam (see Gibson and Sachau, 2000). Thus, sandbaggers are aware of their own abilities, but present oneself as weaker or less capable (Gibson et al., 2002; Petersen, 2014). Lowering others’ expectations of one’s own performance can provide a self-regulation advantage by reducing the pressure to perform (e.g., Baumeister et al., 1985) and the anxiety to fail. This assumption is supported by findings of Gibson and Sachau (2000) showing that individuals scoring higher on the Sandbagging Scale claimed low performance expectations in a computer task only when they were observed by the experimenter and the experimenter signaled to expect high performance from them (performance pressure condition), whereas the sandbagging scores were unrelated to performance predictions when no performance pressure was exerted. Moreover, sandbagging might serve the purpose of setting a low expectation base of one’s own performance in other individuals, in order to exceed the expectation of these others in the following (Gibson and Sachau, 2000; Brown, 2006). For example, Gibson and Sachau (2000) also observed that sandbaggers underestimated their test performance in an upcoming test only when the audience did not have access to their previous test scores. The authors concluded that sandbagging behavior can have a positive effect on others’ performance impressions, and sandbaggers may actively manipulate the environment for personal benefit. As found by Barnett et al. (2018), narcissists appear to engage in more sandbagging behavior which according to these authors may result from their fragile self-esteem and their hypersensitivity to evaluation. The question arises whether and to what extent strategic sandbagging overlaps with IP. A strong association between IP and sandbagging would provide further evidence that the negative self-report of individuals with impostor feelings could be more strategically motivated, for example, to gain interpersonal benefits and that the conceptualization of the IP as an individual’s true doubt about own abilities might need to be reconsidered.

## Research Aims

First of all, based on the mixed results pertaining to gender, the current research aimed to contribute to the question whether the IP shows gender-specific differences. If the finding of higher IP values for women than for men (e.g., Patzak et al., 2017) could be replicated, we further aimed to investigate potential underlying factors of this relationship. Because women compared to men show higher neuroticism scores and lower self-esteem (Vianello et al., 2013; Bleidorn et al., 2016) especially in late adolescence (Zuckerman et al., 2016) and because these traits are assumed to be key correlates of the IP (e.g., Chrisman et al., 1995; Vergauwe et al., 2015), we hypothesized that gender affects IP indirectly through neuroticism and self-esteem. Support for the relevance of this assumption can be also drawn from a recent study of Fassl et al. (2020) who investigated the relationship between gender typing and the IP and found that impostor feelings are not *per se* related to feminine characteristics but rather to the self-ascription of attributes constituting “negative femininity,” which closely resemble neuroticism. Thus, they conclude that it would be worth investigating the relationship between gender and neuroticism and its association to the impostor phenomenon in future studies.

Second, our study sought to further determine the nomological network of the IP. Based on previous research, we expected IP to be negatively related to self-esteem and positively related to neuroticism and perfectionism. With respect to perfectionism, we were particularly interested to elucidate whether the perfectionism of impostors arise from socially prescribed or from self-oriented perfectionism. Since those with impostor feelings seek to be competent and successful in order to gain respect and admiration from others (Ferrari and Thompson, 2006), we assumed that individuals with impostor feelings especially exhibit socially prescribed perfectionism.

Third, the relationship with the sandbagging phenomenon was investigated. As sandbaggers are thought to be aware of their own abilities (Gibson et al., 2002; Petersen, 2014) but present themselves as weaker or less capable in order to establish a low expectation base in others (Gibson et al., 2002; Petersen, 2014), a strong positive relationship between IP and sandbagging would further support the work of Leary et al. (2000) and Leonhardt et al. (2017) showing that IP may also represent strategic aspects.

## Materials and Methods

### Participants and Procedure

The sample comprised 209 participants. The average age of the sample was 26.99years (*SD*=9.96years, range: 18–72years). Gender was measured with the question “Which gender do you feel you belong to?” Participants identified themselves as male (21%, coded as 1) or female (79%, coded as 2). No one indicated feeling like both (coded as 3) or being neither male nor female (coded as 4). The sample had the following educational background: 61.7% were university students (32%) or had a university degree (29.7%), 11.5% had professional education, 22.5% completed Abitur, which is a German school certificate similar to U.S. high school, and 4.3% had less education than abitur. Apart from the third of students, the sample consisted of 49.8% employed individuals and 18.2% persons without employment.

The survey was administered online *via* Unipark (QuestBack). The link to the survey was distributed *via* the university’s and the authors’ information and social media platforms. In total, the link was accessed 448 times and completed 209 times, which corresponds to a completion rate of 46.2%. At the beginning of the survey, the participants were informed about the aim of the study and the conditions for participation. The study protocol was in accordance with the ethical guidelines of the declaration of Helsinki (revised version). Voluntary participation and anonymity were guaranteed, and individuals were asked to give informed consent by clicking the continue button. The online questionnaire started with questions to sociodemographics. Afterward, the measures described in the following were given in the respective order. Further personality questionnaires were assessed that, however, are not relevant for the present study. The survey lasted at average 21.28min (*SD*=13.60, 10 cases were excluded as these individuals interrupted the survey and continued later in the day or at the next days).

### Measures

First, to assess impostor feelings, the German version of the Clance Impostor Phenomenon Scale (Clance, 1985; see also Brauer and Wolf, 2016) was used. The scale comprises 20 items (e.g., “I’m afraid people important to me may find out that I’m not as capable as they think I am”) that had to be answered on a five-point Likert scale ranging from 1 “not applicable at all” to 5 “absolutely correct.” The internal consistency of the scale was excellent (Cronbach’s α=0.92).

Second, sandbagging was measured with the Sandbagging Scale developed by Gibson and Sachau (2000) containing 12 items (e.g., “If I tell others my true ability, I feel added pressure to perform well”) that needed to be answered on a 6-point Likert scale ranging from 1 “I disagree at all” to 6 “I agree exactly.” As no German version of the scale was available at the time of data collection, the English version was translated into German in a team approach (Behr et al., 2015). Initially, three people translated the measure separately. Differences between the three translations were discussed with two bilingual experts, and a solution was worked out for each item followed by the adjudication of the final version. The scale displayed an internal consistency of Cronbach’s α=0.87.

Third, self-esteem was assessed using the German version of the Rosenberg Self-Esteem Scale (RSES; von Collani and Herzberg, 2003), comprising 10 items (e.g., “On the whole I am satisfied with myself”) that had to be answered at a four-point Likert scale ranging from 0 “is absolutely true” to 3 “does not apply at all.” The RSES showed an internal consistency of α=0.91.

Fourth, a German short version (Stöber, 2002) of the Multidimensional Perfectionism Scale (MPS; Hewitt and Flett, 1991) was applied to assess self-oriented perfectionism (8 items, e.g., “I demand nothing less than perfection of myself”) and socially prescribed perfectionism (10 items, e.g., “People expect nothing less than perfection from me”) using a six-point Likert scale ranging from 1 “is not true at all” to 6 “is exactly true.” Internal consistencies were *α*=0.90 for self-oriented perfectionism and *α*=0.85 for socially prescribed perfectionism.

Finally, the five-factor model of personality was assessed by means of a short version of the Big Five Inventory (BFI-K; Rammstedt and John, 2005) with 4 to 5 items for each dimension (e.g., neuroticism: “I worry a lot”) that were answered on a five-point Likert scale ranging from 1 “very inappropriate” to 5 “very appropriate.” Internal consistencies were *α*=0.76 for neuroticism, *α*=0.81 for extraversion, *α*=0.73 for openness to experience, *α*=0.68 for agreeableness, and *α*=0.70 for conscientiousness.

### Statistical Analyses

All analyses were conducted using IBM SPSS Statistics 23 (SPSS Inc., Chicago, IL, United States). Gender-related differences in the IP were examined using *t* tests. The analyses on indirect effects of gender on IP *via* neuroticism and self-esteem were conducted with PROCESS (Hayes, 2018). Indirect effects were estimated by the computation of bias-corrected bootstrap confidence intervals. The null hypothesis of no indirect effect can be rejected at an alpha level of 0.05 if the 95% bootstrap confidence interval (based on 5,000 resamples) does not include zero (Preacher et al., 2007). To investigate personality correlates of the IP, bivariate correlations were calculated and the incremental value of the associated personality factors was assessed *via* linear regression analysis.

A power analysis using G^*^Power (Faul et al., 2007) showed that with our sample size of *N*=209, given an α of 0.05 and a statistical power (1-β error probability) of 0.80, we could detect gender effects of medium size (*d*=0.47) and bivariate correlations of small size (*r*=0.19).

## Results

Research data are available under https://osf.io//q27hp/(the link will be made public after acceptance).

### Descriptives of the IP

IP was normally distributed as indicated by Shapiro-Wilk test, *df*(209)=0.990, *p*=0.165 and visual inspection of histogram and Q-Q plot. The test scores varied between 25 and 94 with a mean of 55.36 and a standard deviation of 14.87. According to the categorization proposed by Clance (1988, but never empirically validated), 17.7% of the sample (*n*=37) reported no or few impostor feelings (*x_i_*<41), 45.0% (*n*=94) reported having moderate impostor feelings (*x_i_*<61), while 32.1% (*n*=67), and 5.3% (*n*=11) reported frequent (*x_i_*<81) and intense (*x_i_*>80), respectively, impostor feelings.

### Gender-Related Differences in the IP

**Figure 1:**
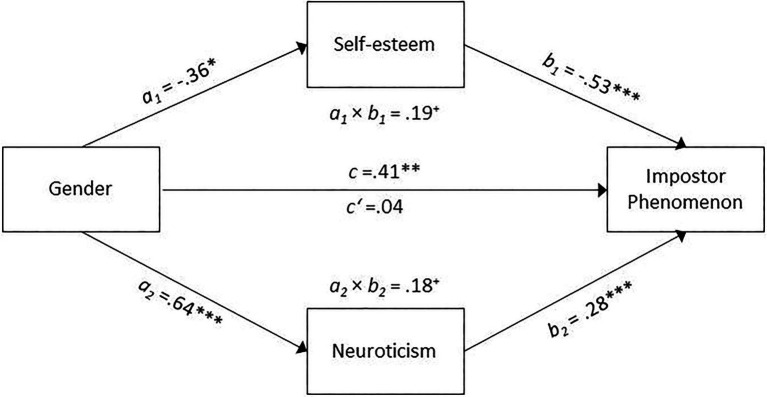
Indirect effect of gender on the impostor phenomenon *via* self-esteem and neuroticism and controlled for age. That the standardized coefficients are reported. ^*^p<0.05, ^**^p<0.01, ^***^p<0.001, ^+^significant according to the 95% bootstrapping confidence interval.

IP was normally distributed in both gender groups (Shapiro-Wilk test: *p*_male_=0.17, *p*_female_=0.49), and the assumption of homogeneity of variances was also given (*p*=0.826). As indicated by a significant *t* test, *t*(207)=−3.44, *p*=0.001, Hedge’s *g*=−0.58 (95% *CI*=−0.25 to −0.92), female participants (*M*=57.15, *SD*=14.44) reported higher impostor feelings than males (*M*=48.68, *SD*=14.68). Next, we aimed to examine whether gender might indirectly affect IP *via* self-esteem or neuroticism. We additionally considered age as potential covariate because males (*M*=33.66, *SD*=13.45) were significantly older than females (*M*=25.21, *SD*=7.95) in our sample, *t*(207)=3.99, *p*<0.001, Hedge’s *g*=0.90 (95% *CI*=0.56 to 1.25). Accordingly, a parallel mediation model (see Figure 1) was conducted, where self-esteem and neuroticism were considered as mediator variables and age as covariate. Gender was significantly associated with both, self-esteem, *a_2_*=−0.36, *p*=0.045, and neuroticism, *a_2_*=0.64, *p*<0.001, with females reporting lower self-esteem and higher neuroticism than males (self-esteem: *M/SD* =21.43/6.57 for females vs. 23.98/5.23 for males; neuroticism: *M/SD*=3.41/0.89 for females vs. 2.73/0.90 for male). There was no significant direct effect of gender on IP, *c′*=0.04, *p*=0.761. Both indirect effects were significant and of similar size, self-esteem: *a_1_b_1_*=0.19, 95% bootstrap *CI*=0.02 to 0.37; neuroticism: *a_2_b_2_*=0.18, 95% bootstrap *CI*=0.07 to 0.31. The estimated value of the total indirect effect was *a_1_b_1_ +a_2_b_2_*=0.37, 95% bootstrap *CI*=0.14 to 0.60.

### Personality Correlates and Discriminant Validity of the Impostor Construct

The intercorrelations of the IP measure with all personality measures considered and the descriptives of these measures are depicted in Table 1. IP and sandbagging were highly associated (*r*=0.73, *p*<0.001). A similar association with IP was observed for self-esteem (*r*=−0.71, *p*<0.001) and neuroticism (*r*=0.62, *p*<0.001). Small to moderate negative associations were observed for agreeableness (*r*=−0.18, *p*<0.01), extraversion (*r*=−0.20, *p*<0.01), and conscientiousness (*r*=−0.33, *p*<0.001). With respect to perfectionism, IP showed a stronger positive correlation with socially prescribed perfectionism (*r*=0.52, *p*<0.001) than with self-related perfectionism (*r*=0.19, *p*<0.01). This difference in correlation coefficients was significant (*Fisher’s z*=3.90; *p*<0.001).

**Table 1:** Descriptives and intercorrelations of the demographic and personality variables.

	*M*	*SD*	1	2	3	4	5	6	7	8	9	10	11	12
1. Gender	–	–	–	−0.35^***^ [−0.46, −0.23]	0.23^***^ [0.10, 0.35]	0.21^**^ [0.08, 0.34]	0.11 [−0.03, 0.24]	−0.03 [−0.17, 0.11]	−0.16^*^ [−0.29–0.02]	0.30^***^ [0.17, 0.42]	0.07 [−0.07, 0.20]	0.03 [−0.11, 0.17]	−0.03 [−0.14, 0.14]	0.14^*^ [0.01, 0.27]
2. Age	26.99	9.96		–	−0.25^***^ [−0.37, −0.12]	−0.29^***^ [−0.41, −0.16]	−0.02 [−0.16, 0.12]	0.06 [−0.08, 0.19]	0.10 [−0.04, 0.23]	−0.21^**^ [−0.34, −0.08]	−0.07 [−0.20, 0.07]	−0.08 [−0.21, 0.06]	−0.01 [−0.15, 0.13]	0.15^*^ [0.01, 0.28]
3. Impostor	55.36	14.87			**0.92**	0.73^***^ [0.66, 0.79]	0.19^**^ [0.06, 0.32]	0.52^***^ [0.41, 0.61]	−0.71^***^ [−0.77, −0.64]	0.62^***^ [0.53, 0.70]	−0.20^**^ [−0.33, −0.07]	0.09 [−0.05, 0.22]	−0.18^**^ [−0.31, −0.05]	−0.33^***^ [−0.45, −0.20]
4. Sandbagging	3.64	0.82				**0.87**	0.25^***^ [0.12, 0.37]	0.39^***^ [0.27, 0.50]	−0.48^***^ [−0.58, −0.37]	0.41^***^ [0.29, 0.52]	−0.13 [−0.26, 0.01]	0.10 [−0.04, 0.23]	−0.11 [−0.24, 0.03]	−0.21^**^ [−0.34, −0.08]
5. Self. Perfectionism	3.99	1.01					**0.90**	0.32^***^ [0.19, 0.44]	−0.12 [−0.25, 0.02]	0.15^*^ [0.01, 0.28]	0.02 [−0.12, 0.16]	0.14^*^ [0.01, 0.27]	−0.07 [−0.20, 0.07]	0.40^***^ [0.28, 0.51]
6. Soc. Perfectionism	2.46	0.73						**0.85**	−0.53^***^ [−0.62, −0.42]	0.25^***^ [0.12, 0.37]	−0.18^**^ [−0.31, −0.05]	0.04 [−0.10, 0.17]	−0.14^*^ [−0.27, −0.01]	−0.15^*^ [−0.28, −0.01]
7. Self-esteem	21.97	6.39							**0.91**	−0.57^***^ [−0.66, −0.47]	0.23^**^ [0.10, 0.35]	−0.15^*^ [−0.28, −0.01]	0.17^*^ [0.04, 0.30]	0.28^***^ [0.15, 0.40]
8. Neuroticism	3.27	0.93								**0.76**	−0.18^**^ [−0.31, −0.05]	0.22^**^ [0.09, 0.35]	−0.12 [−0.25, 0.02]	−0.23^***^ [−0.35, −0.10]
9. Extraversion	3.51	0.90									**0.81**	0.22^**^ [0.09, 0.35]	0.11 [−0.03, 0.24]	0.17^*^ [0.04, 0.30]
10. Openness	3.95	0.75										**0.73**	0.05 [−0.09, 0.18]	0.09 [−0.05, 0.22]
11. Agreeable ness	2.96	0.85											**0.68**	0.12 [−0.02, 0.25]
12. Conscientious ness	3.68	0.72												**0.70**

*Mean (M), standard deviation (SD), and intercorrelations of the personality variables*.

* *p<0.05;*

** *p<0.01;*

*** *p<0.001; N=209.*

*Cronbach’s α of the scales is given at the diagonal in bold. The 95% confidence intervals are given in parentheses*.

In a next step, regression analyses were conducted to examine the incremental value of the related personality variables in explaining variance in the IP. Given the fact that IP and sandbagging were highly associated (*r*=0.73, *p*<0.001) and that sandbagging showed similar associations with the addressed personality variables (see Table 1), it was further examined whether IP and sandbagging could indeed be considered as different constructs. To address this issue, we aimed to investigate whether the observed associations with the personality variables are more specific for one or the other construct. Therefore, two hierarchical regression models were conducted, where either IP (model 1) or sandbagging (model 2) was regressed on age, gender, and the personality variables that were significantly associated with IP or/and sandbagging in the first step of the regression. In the second step, sandbagging (model 1) and IP (model 2), respectively, were included as predictor to investigate which associations remained significant when controlling for the respective other factor.

In model 1 considering IP as dependent variable (see Table 2, left columns), four of the seven personality variables significantly explained variance in the criterion. Neuroticism (*β*=0.25, *p*<0.001) and socially prescribed perfectionism (*β*=0.21, *p*<0.001) were positively related, whereas self-esteem (*β*=−0.37, *p*<0.001) and conscientiousness (*β*=−0.15, *p*=0.004) were negatively related to the IP measure. Using Bonferroni-corrected *p*-values that take the number of predictors in the model into account (*p*<0.05/9=0.0055), all these effects would remain significant. When sandbagging was additionally included in the model, the corrected *R^2^* significantly improved from 0.63 to 0.75 (*p*<0.001). The personality effects all remained significant at the conventional significance level and except of conscientiousness would even hold a conservative Bonferroni correction (*p*<0.05/10=0.005). Sandbagging explained the largest amount of variance in the IP (*β*=0.41, *p*<0.001), followed by self-esteem (*β*=−0.28, *p*<0.001), neuroticism (*β*=0.21, *p*<0.001), and socially prescribed perfectionism (*β*=0.14, *p*=0.002).

**Table 2:** Incremental validity of trait factors significantly associated with the impostor phenomenon and sandbagging behavior.

	Model 1			Model 2	
Criterion	Impostor phenomenon			Sandbagging behavior	
Step	*β*	*p*		*β*	*p*
1. Age	−0.13	0.008		−0.21	0.001
Gender	0.07	0.151		0.07	0.273
Self-esteem	−0.37	<0.001		−0.23	0.005
Neuroticism	0.25	<0.001		0.11	0.133
Extraversion	−0.01	0.771		−0.02	0.714
Agreeableness	−0.04	0.365		−0.01	0.981
Conscientiousness	−0.15	0.004		−0.15	0.039
Self-oriented perfectionism	0.09	0.092		0.21	0.003
Socially prescribed perfectionism	0.21	<0.001		0.17	0.021
***R***^**2**^ ***corrected***	**0.63**	**<0.001**		**0.35**	**<0.001**

2. Age	−0.04	0.319		−0.12	0.028
Gender	0.04	0.318		0.02	0.709
Self-esteem	−0.28	<0.001		0.04	0.588
Neuroticism	0.21	<0.001		−0.07	0.259
Extraversion	−0.01	0.913		−0.01	0.804
Agreeableness	−0.04	0.289		0.03	0.579
Conscientiousness	−0.09	0.041		−0.04	0.547
Self-oriented perfectionism	0.01	0.923		0.14	0.017
Socially prescribed perfectionism	0.14	0.002		0.01	0.824
Sandbagging	0.41	<0.001	IP	0.72	<0.001

***R***^**2**^ ***corrected***	**0.75**	**<0.001**		**0.54**	**<0.001**
***change in F***	**82.61**	**<0.001**		**82.61**	**<0.001**

*In regression model 1, the impostor phenomenon (left columns), and in regression model 2, the sandbagging construct (right columns) was regressed on age, gender, and the personality factors self-esteem, neuroticism, extraversion, agreeableness, conscientiousness, self-oriented perfectionism, and socially prescribed perfectionism in step 1 of the regression. In step 2, additionally the sandbagging (model 1) and the impostor phenomenon (IP), respectively, was included (N=209)*.

Regarding model 2 with sandbagging as dependent variable (see Table 2, right columns), also four of the seven personality variables explained variance in the sandbagging construct. Similar to IP, self-esteem (*β*=−0.23, *p*=0.005) and conscientiousness (*β*=−0.15, *p*=0.039) were negatively, whereas socially prescribed perfectionism (*β*=0.17, *p*=0.021) was positively related to sandbagging. Moreover, self-related perfectionism (*β*=0.21, *p*=0.003) explained variance in sandbagging. When IP was included at the second step of model 2, the corrected *R^2^* significantly improved from 0.35 to 0.54 (*p*<0.001). IP significantly explained variance in sandbagging (*β*=0.72, *p*<0.001), while the prediction of almost all personality variables got insignificant. Only self-oriented perfectionism (*β*=0.14, *p*=0.017) remained significant at the conventional significance level with a small effect size, but not at the Bonferroni-corrected one (*p*<0.05/10=0.005). In sum, these analyses demonstrate that low self-esteem, high neuroticism, and socially prescribed perfectionism appear to be rather specific for IP than for sandbagging.

## Discussion

In this study, we aimed to contribute to a better understanding of the IP by addressing the following questions: (1) Do female participants report higher IP scores than male participants and are there indirect effects *via* self-esteem and neuroticism as key correlates of the IP? (2) Is the IP linked to a self-oriented, intrinsic perfectionism or rather to perfectionism due to social expectations? (3) Could the negative self-presentation of impostors be due to more strategic reasons than to the actual conviction of being intellectually incapable? In addition, the incremental validity of the considered personality correlates in explaining variance in the IP was of interest. In the following sub-sections regarding gender differences in the IP and the nomological network of the IP, we will discuss these questions and will also highlight limitations and future directions of our study.

### Gender-Related Differences in the IP

In this study, women reported significantly higher IP scores than men, which is in line with the initial assumption of Clance and Imes (1978) that the IP is a female-specific phenomenon and also converges with several studies suggesting higher scores for women, whereas other studies did not find gender-specific differences in the IP (for an overview see Bravata et al., 2019). To deepen the understanding of the gender-specific effect, we conducted an additional analysis looking at gender effects on IP *via* self-esteem and neuroticism, as neuroticism and self-esteem have been consistently associated with IP and are considered key correlates of the IP (e.g., Chrisman et al., 1995; Thompson et al., 2000; Vergauwe et al., 2015; Schubert and Bowker, 2019) and have also be shown to differ between gender (Weisberg et al., 2011; Vianello et al., 2013; Bleidorn et al., 2016; Zuckerman et al., 2016). Indeed, both the higher neuroticism and lower self-esteem of women compared to men contributed to gender-related differences in the IP. Notably, this indirect effect does not depend on the age-related differences in our sample, as age was included as covariate in the model.

It has to be noted, however, that cross-sectional designs limit strong causal inference of results, such as compared to highly controlled experimental designs. However, with gender as the independent variable, at least the direction of the independent variable and neuroticism and self-esteem as potential mediators can be considered rather uncritical in terms of temporality in our model. With respect to the question whether IP shows gender effects or not, future research might also systematically assess moderators of the gender – IP relationship, such as situational context factors that might increase or decrease impostor feelings. For example, to be faced with stereotype threats, that is the fear of doing something that would inadvertently confirm gender stereotypes, might drive gender-specific differences in the IP. Studies on IP in minority groups, for instance, suggest that being the first in their families to pursue advanced education may lead to impostor feelings (Peteet et al., 2015). In addition, Badawy et al. (2018) showed that male and female imposters differ with respect to situational factors leading to impaired performance and anxiety. In their study, only male imposters were significantly affected by negative feedback and conditions with high accountability, probably affecting their competence-based self-views and their self-esteem. That is, context factors that may drive differences between women and men should be taken into account in future studies to better understand the inconsistently observed gender differences in the IP (for a detailed discussion on the importance of contextual factors in understanding the IP, see Feenstra et al., 2020).

A further limitation with respect to answering the question of gender differences in the IP was the unequal gender distribution in our study. However, as all *t* test assumptions (normality of the IP in both groups, homogeneity of variances) were given, as we used effect size coefficient that accounts for different sample sizes in both groups, and as unequal sample size should rather increase the probability of Type I than Type II error (Rusticus and Lovato, 2014), we are convinced that the found gender-related differences in the IP should be quite robust in our sample.

### Personality Correlates and Discriminant Validity of the IP

With respect to the personality-related aspects of the IP, we aimed to replicate the frequently reported associations with neuroticism, self-esteem, and perfectionism, but specifically aimed to unravel whether the IP is rather related to socially prescribed perfectionism or to perfectionism due to high expectations of oneself. Moreover, we were particularly interested whether IP is associated with (strategic) sandbagging behavior that is conceptually similar with respect to the behavioral output (claiming to be less than one is), but differs regarding the assumed awareness the individual has of his/her own abilities.

In line with previous research (e.g., Vergauwe et al., 2015; Schubert and Bowker, 2019), relatively strong associations were observed between the IP and self-esteem, neuroticism, and perfectionism, respectively, which were preserved when controlling for the variances of the other variables in a multiple regression analysis. Considering the incremental value of each trait factor, especially a low self-esteem was related to the IP, followed by neuroticism, and socially prescribed perfectionism.

Regarding perfectionism, our results particularly suggest a relationship between IP and the sub-dimension socially prescribed perfectionism of the MPS of Hewitt and Flett (1991). This dimension describes the belief that others expect perfection from oneself and has been consistently found to relate to negative outcomes and psychological malfunctioning and poor adjustment such as negative affect, anxiety, depression, shame, and guilt as well as a low self-esteem and maladaptive coping styles (e.g., Klibert et al., 2005; Jahromi et al., 2012). Further, socially prescribed perfectionism is associated with perfectionistic concerns (see Gäde et al., 2017), one of two higher order factors in models of multidimensional perfectionism that is linked to concerns over mistakes, doubts about actions and fear of failure (for an overview, see Stoeber and Otto, 2006). In contrast, the sub-dimension self-oriented perfectionism, conceptualized as demanding perfection of oneself, was only weakly associated with IP and showed no incremental validity over and above the other personality variables in the present study. This dimension is related to the higher order factor perfectionistic striving and has been rather associated with adaptive aspects, such as positive affect, assertiveness, resourcefulness, and intrinsic motivation (Klibert et al., 2005; Stoeber and Otto, 2006). Thus, our results support previous assumptions that impostors are afraid of negative evaluations and strive to meet social expectations (Thompson et al., 2000) by showing that they are less perfectionist and self-critical out of their own demands, but above all have the feeling that they have to meet perfectionist demands of their social environment. This is also in line with findings of Leary et al. (2000) that individuals with high impostor feelings only tended to a negative self-presentation when their performance was public. The result pattern further shows that IP is especially related to maladaptive aspects of perfectionism and thus supports recent findings of Vergauwe et al. (2015).

In addition, it was investigated to what extent IP can be distinguished from the sandbagging construct. Sandbagging is conceived as the tendency to initially present oneself as worse than one is for strategic reasons, for example, in order to keep the expectations of others low and to surprise positively with one’s own performance (Gibson et al., 2002; Petersen, 2014). We observed a large overlap between IP and sandbagging. Given, the observed correlation of *r*=0.73, both constructs shared 53% of their variance. Nevertheless, IP in particular still seems to hold aspects that go beyond sandbagging because the significant correlations between sandbagging and personality factors largely disappeared when IP was included as additional predictor in a stepwise linear regression. Here, only self-oriented perfectionism showed up as incrementally valid. On the other hand, significant associations between personality and IP were largely preserved when sandbagging was included in the regression model. Thus, despite the large overlap, our results do not support a Jangle Fallacy, that is the wrong assumption that two scales with different names measure different constructs (Kelley, 1927). Nevertheless, the high association of IP and sandbagging may support previous results by Leary et al. (2000) and Leonhardt et al. (2017) showing that for at least some of the individuals with impostor feelings their negative self-representation may be less a consequence of genuine self-doubt about one’s intellectual abilities but more strategically motivated. Thus, as with sandbagging, setting a low expectation base with others could be one relevant motive for individuals with feelings of impostor.

With respect to our aim to further investigate the nomological network of the IP two limitations have to be discussed. First, with *N*=209, our sample size was somewhat lower than the N≥250 recommended by Schönbrodt and Perugini (2013) for the field of personality research. This recommendation was based on an assumed effects size of *r*=0.21. In our study, however, we were interested in the nomological network of the IP. Here, the expected correlations were of moderate to large size and Schönbrodt and Perugini (2013) argue that in these contexts also smaller sample sizes might be adequate. We further used the very conservative Bonferroni correction to limit alpha error accumulation and to focus on the core constructs associated with IP. Thus, our interpreted effects should be essentially robust even if we slightly missed the recommended sample size of Schönbrodt and Perugini (2013).

Second, our sample was heterogeneous with respect to the occupational status, that is it consisted of professionals, university students, and unemployed individuals. Due to the overall sample size, however, it was not possible to investigate whether the associations between IP and the considered personality variables were invariant across these occupational status groups. Because previous research indicates that IP is less pronounced in professionals than students and associations with external variables differ between these groups (Neureiter and Traut-Mattausch, 2016; Brauer and Proyer, 2017, 2019), future research should investigate the role of occupational status as moderator of the reported associations. Future research may further assess objective measure of achievement (e.g., grades or other indicators of academic or occupational success) and investigate whether high and low achievers with impostor feelings differ with respect to the nomological network of the IP.

## Conclusion

With this study, we intended to contribute to several open questions regarding gender differences in and the nomological network of the IP. Evidence was found that key correlates of the IP, that is, lower self-esteem and higher neuroticism, may also play a crucial role in explaining why women report higher impostor feelings than men in many studies. Moreover, a strong association with socially prescribed perfectionism, which is related to the belief that others expect perfection from one, but not to the self-oriented perfectionism of the Multidimensional Perfectionism Scale, underscores that the negative self-presentation of impostors may be primarily motivated by others. This is also supported by the incremental relationship given with the sandbagging construct. Similarly to IP, “sandbaggers” portray themselves negatively to others, but do so for very strategic reasons to create a low expectancy base in others, possibly for the purpose of gaining interpersonal advantages. This, in turn, would challenge the conceptualization of the IP as a genuine doubt about one’s own competencies. The present research further contributes to our understanding of the impostor phenomenon and may stimulate future research which could use (quasi) experimental designs for example to investigate the contextual moderating factors that promote impostor feelings and that motivate individuals to present themselves negatively toward others.

## Data Availability Statement

Research data are available under https://osf.io/q27hp/.

## Ethics Statement

All participants were informed about the aim of the study and the conditions for participation. The study protocol was in accordance with the ethical guidelines of the Declaration of Helsinki (revised version). Voluntary participation and anonymity were guaranteed, and informed consent was given for participation in this study.

## Author Contributions

JW, SE, and LM conceived and designed the study. JW collected the data and MF and SE analyzed and interpreted the data. MF, SE, LM, and JW wrote or commented on the paper. MF revised the manuscript allong the reviewers’ comments. All authors contributed to the article and approved the submitted version.

## Conflict of Interest

The authors declare that the research was conducted in the absence of any commercial or financial relationships that could be construed as a potential conflict of interest.

## Publisher’s Note

All claims expressed in this article are solely those of the authors and do not necessarily represent those of their affiliated organizations, or those of the publisher, the editors and the reviewers. Any product that may be evaluated in this article, or claim that may be made by its manufacturer, is not guaranteed or endorsed by the publisher.
